# Vascular endothelial growth factor gene polymorphisms and renal cell carcinoma: A systematic review and meta-analysis

**DOI:** 10.3892/ol.2013.1499

**Published:** 2013-07-29

**Authors:** YONG ZHANG, SHENG LI, HOU-QIN XIAO, ZHAO-XIONG HU, YAN-CHENG XU, QI HUANG

**Affiliations:** 1 Department of Endocrinology, Zhongnan Hospital, Wuhan University, Wuhan, Hubei 430071, P.R. China; 2 Department of Nephropathy, Taihe Hospital, Hubei University of Medicine, Shiyan, Hubei 442000, P.R. China; 3 Department of Urology, Zhongnan Hospital, Wuhan University, Wuhan, Hubei 430071, P.R. China

**Keywords:** vascular endothelial growth factor, polymorphism, renal cell carcinoma, risk, meta-analysis

## Abstract

Renal cell carcinoma (RCC) accounts for 3% of all cancer-related mortalities in adults. The risk factors for the development of RCC remain under investigation. Vascular endothelial growth factor (VEGF) is a key mediator of angiogenesis and is crucial for the development and metastasis of tumors, including RCC. VEGF gene polymorphisms may alter VEGF protein concentrations, affect the process of angiogenesis and may be involved in inter-individual variation in carcinogenesis. In the present study, a systematic review and meta-analysis were performed based on published case-control studies in order to estimate the association between VEGF gene polymorphisms and the susceptibility to RCC. A total of five studies that involved eight polymorphisms and were published between January 2000 and December 2012 were identified from PubMed. The results of this systematic review and meta-analysis indicate that the VEGF 936C/T, 1612G/A, −1154G/A, −2549I/D, −460T/C and 405G/C gene polymorphisms are not associated with the risk of RCC. There was no polymorphism in 702C/T and RCC and the −2578C/A gene polymorphism may be associated with an increased risk of RCC. However, due to the limitations of the present study, further high quality case-control studies are warranted to confirm these findings.

## Introduction

Renal cell carcinoma (RCC) is the seventh most common cancer in males and the ninth most common cancer in females, and accounts for ~2% of all malignant diseases in adults (1). RCC continues to be a devastating cancer and the worldwide incidence and mortality rates are rising at a rate of 2–3% per decade (2). Furthermore, the initial clinical course of RCC is asymptomatic, resulting in 25–30% of patients presenting with metastatic disease at the time of diagnosis (2). To date, the definite etiology of RCC remains unclear. A number of studies have shown that active and/or passive smoking (3), moderate and/or heavy alcohol consumption (4), obesity (5,6) and hypertension (7) are established risk factors that play key roles in the development of RCC. However, as they do not entirely explain the etiology, there may be other risk factors that are involved. With developments in molecular biology, researchers have investigated whether genetic factors are involved in RCC development.

Vascular endothelial growth factor (VEGF) is a potent endothelial cell mitogen that plays a key role in angiogenesis (8,9). Compelling evidence from meta-analyses has indicated that VEGF gene polymorphisms are associated with the risk of various diseases, including gastric cancer (10), pre-eclampsia (11), cardiovascular disease (12) and amyotrophic lateral sclerosis (13). The VEGF receptors, VEGFR1, VEGFR2 and VEGFR3, have significant roles in the signaling pathways that are involved in RCC pathogenesis, and mutations in VEGFRs may affect the signaling networks (14). Therefore, VEGF gene polymorphisms may be associated with RCC. In 2002, Abe *et al*(15) investigated the association between single nucleotide polymorphisms in the 3′-untranslated region (UTR) of the VEGF gene and RCC in the Japanese population, and indicated that the C702T, C936T and G1612A polymorphisms in the 3′-UTR of the VEGF gene are not associated with the risk of RCC. However, their results showed significant ethnic differences in the frequencies of the C702T and G1612A alleles (15).

Certain studies have also been performed to detect the association between VEGF gene polymorphisms and RCC, and the results are varied. The present systematic review and meta-analysis aims to present the genetic knowledge on the VEGF gene polymorphisms and RCC risk in humans based on the published evidence.

## Materials and methods

### Literature selection

The proposed Preferred Reporting Items for Systematic reviews and Meta-Analyses (PRISMA) (16) statement was followed to report the present meta-analysis. Initially, the published studies that tested the association between VEGF gene polymorphisms and RCC were identified by searching PubMed for studies that were published between January 2000 and December 2012. The search terms that were used were ‘renal cell’ and ‘carcinoma’ or ‘cancer’ and ‘vascular endothelial growth factor’ or ‘VEGF’ and ‘polymorphism’, ‘mutation’ or ‘variation’, without restrictions. In addition, the reference lists of retrieved papers and recent reviews were also examined.

### Study selection

Any study that matched the following criteria was included: i) A case-control study design; ii) an association between VEGF gene polymorphisms and RCC in humans; iii) RCC confirmed by the accepted diagnostic criteria; iv) inclusion of the odds ratio (OR) and the corresponding 95% confidence intervals (CIs), or the number of events that may be used to calculate them.

To evaluate the eligibility of all the studies retrieved from the databases on the basis of the predetermined selection criteria, two independent investigators were used. Disagreements were resolved by discussion.

### Data extraction

The two independent reviewers extracted the following data: gene polymorphisms, first author’s last name, year of publication, site of origin, source of controls, matching criteria, number of cases and controls, number of different genotypes in cases and controls, Hardy-Weinberg equilibrium (HWE) and minor allele frequency in controls. Any disagreements were resolved by consensus.

### Data analysis

The articles that met the inclusion criteria were identified and classified according to the gene polymorphisms that they described. Subsequent to categorizing the data, a descriptive analysis of the data that were unsuitable for conducting the meta-analysis was performed. The data that were suitable for pooling were used in the meta-analysis.

A pooled OR and 95% CI was computed for the risk allele using RevMan 5.1 (Nordic Cochrane Centre, Copenhagen, Denmark) to generate forest plots, in order to determine whether a statistical association was present between the case and control groups and to assess the heterogeneity of the included studies. The HWE was tested by a χ^2^ test at a significance level of P<0.05. Heterogeneity was quantifiably evaluated using the χ^2^-based Cochran’s Q statistic (17) and the I^2^ statistic (18). The I^2^ statistic yields results ranging between 0 and 100% (0–25%, no heterogeneity; 25–50%, moderate heterogeneity; 50–75%, large heterogeneity; and 75–100%, extreme heterogeneity). If heterogeneity was present, the random effects model was used, otherwise, the fixed effects model was used. A sensitivity analysis was conducted by switching the effects models. If possible, potential publication bias was assessed by a visual inspection of the funnel plots.

## Results

### Identification of eligible studies

**Figure 1:**
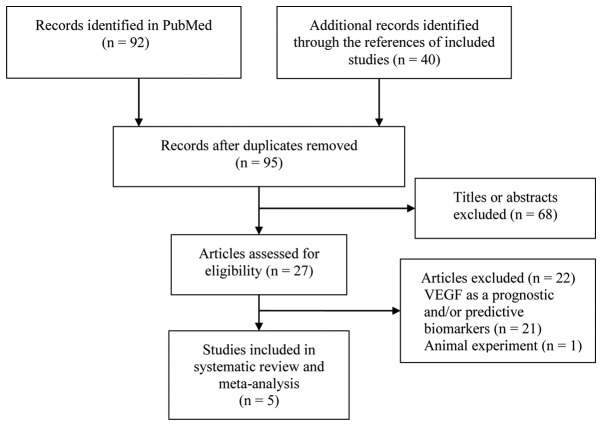
Flow diagram of the study selection process.

Of the initial 132 references, five case-control studies (15,19–22) were identified, including eight polymorphisms. A flow chart showing the study selection process is presented in Fig. 1.

### Characteristics of studies

The detailed characteristics of the included studies are summarized in Tables I and II. All studies were published in English and the sample sizes ranged between 51 and 343 participants. The controls were all healthy individuals and were matched for age and gender. The genotypes of two studies (19,20) were analyzed using PCR (polymerase chain reaction), two (15,21) were analyzed by PCR-RFLP (restriction fragment length polymorphism) and one (22) by a TaqMan assay. The genotype distributions in the controls of all the studies were in accordance with the HWE.

Abe *et al*(15) studied three polymorphisms, 936C/T (rs3025039), 1612G/A (rs10434) and 702C/T. The study by Ricketts *et al*(19) was concerned with one polymorphism, −1154G/A (rs1570360). Bruyère *et al*(20) investigated five polymorphisms, 936C/T (rs3025039), −1154G/A (rs1570360), −2549I/D, −460T/C (rs833061) and 405G/C (rs2010963). Ajaz *et al*(21) surveyed two polymorphisms, −2578C/A (rs699947) and 936C/T (rs3025039). Sáenz-López *et al*(22) inquired about three polymorphisms, 936C/T (rs3025039), −460T/C (rs833061) and −2578C/A (rs699947).

### 936C/T polymorphism and RCC

**Figure 2:**
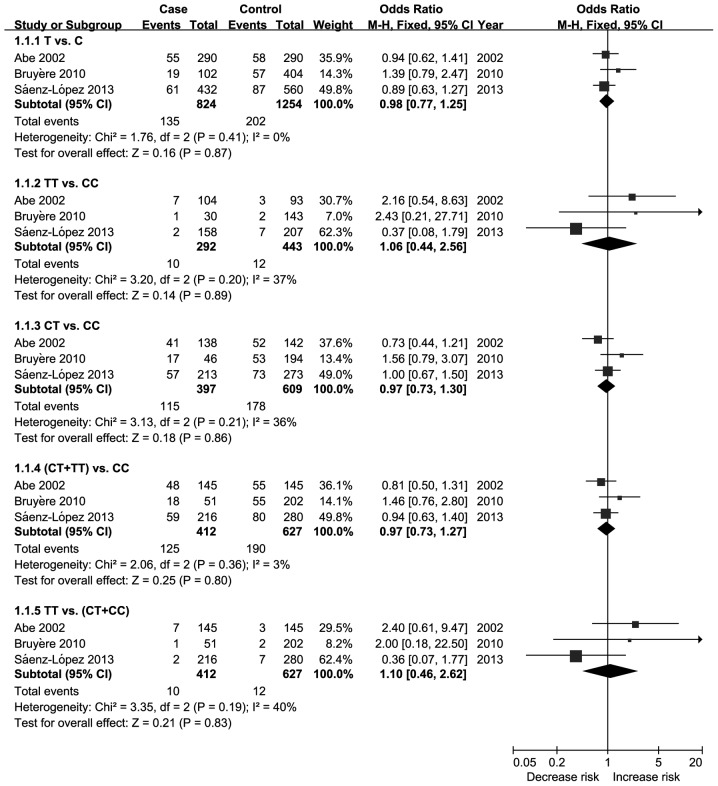
Forest plot of the association between the 936C/T polymorphism and RCC risk based on the fixed effects model. RCC, renal-cell carcinoma; CI, confidence interval.

**Figure 3:**
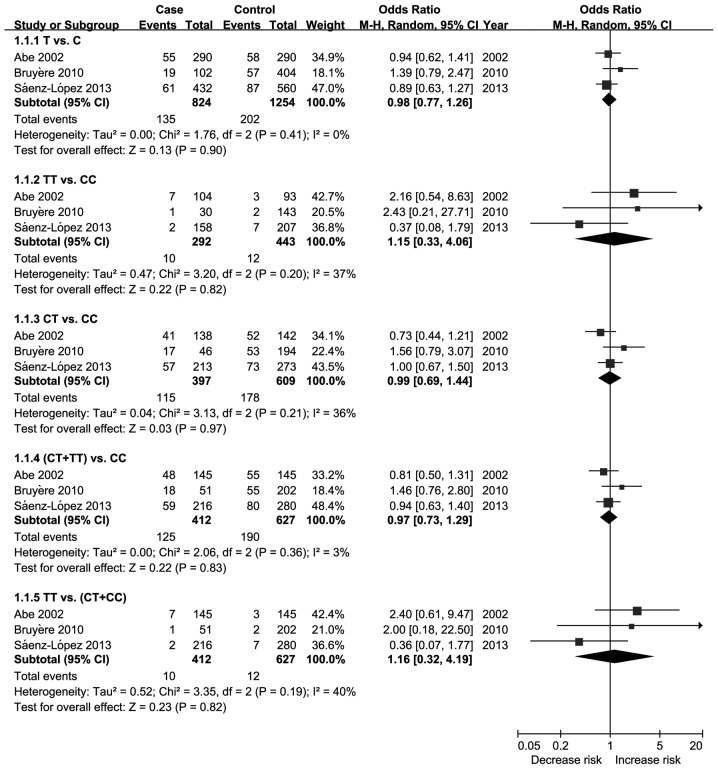
Forest plot of the association between the 936C/T polymorphism and RCC risk based on the random effects model. RCC, renal cell carcinoma, CI, confidence interval.

A total of four studies (15,20–22) investigated the 936C/T polymorphism in RCC. Of these studies, three (15,20,22) provided enough data to be combined. Figs. 2 and 3 show the results of the meta-analysis based on the fixed effects and random effects models, respectively. The results indicate that the VEGF gene 936C/T polymorphism was not associated with the risk of RCC.

Ajaz *et al*(21) did not report the number of each genotype distribution of the VEGF 936C/T polymorphism. They reported that the 936C/T polymorphism lacked an association with RCC (OR, 1.5; 95% CI, 0.7–3.3; P=0.36).

### 1612G/A polymorphism and RCC

Only Abe *et al*(15) investigated the VEGF gene 1612G/A polymorphism and RCC. The results showed no association between the 1612G/A polymorphism and a risk of RCC [A vs. G (OR, 0.83; 95% CI, 0.50–1.36; P=0.45); AA vs. GG (OR, 0.32; 95% CI, 0.03–3.14; P=0.33); AG vs. GG (OR, 0.91; 95% CI, 0.52–1.58; P=0.73); AA+AG vs. GG (OR, 0.86; 95% CI, 0.50–1.48; P, 0.58); AA vs. GG+GA; OR, 0.33; 95% CI, 0.03–3.20; P=0.34)].

### 702C/T polymorphism and RCC

Abe *et al*(15) tested the VEGF gene 702C/T polymorphism and RCC. The results showed that there was no polymorphism in 702C/T in either the case or control groups (data shown in Table II).

### −1154G/A polymorphism and RCC

**Figure 4:**
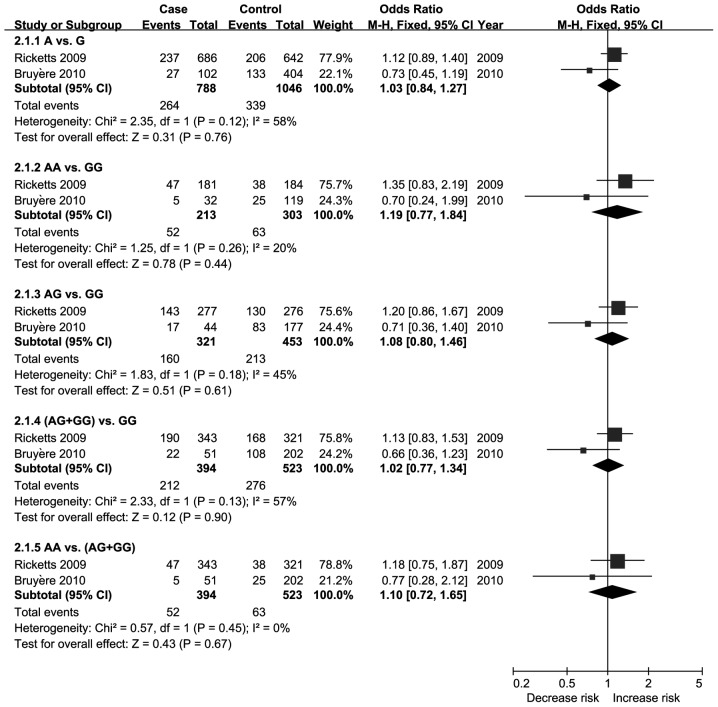
Forest plot of the association between the −1154G/A polymorphism and RCC risk based on the fixed effects model. RCC, renal-cell carcinoma; CI, confidence interval.

**Figure 5:**
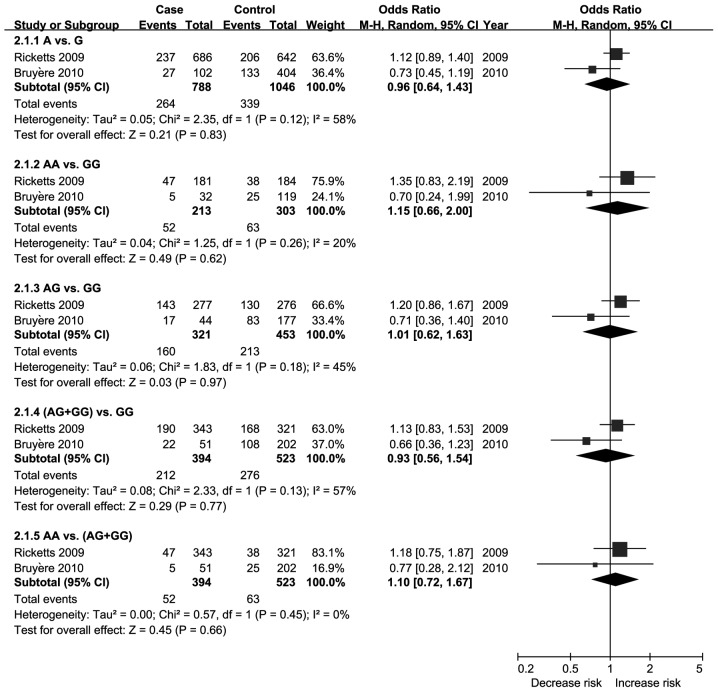
Forest plot of the association between the −1154G/A polymorphism and RCC risk based on the random effects model. RCC, renal-cell carcinoma; CI, confidence interval.

A total of two studies (19,20) investigated the −1154G/A polymorphism and RCC. Figs. 4 and 5 show the results of the meta-analysis based on the fixed effects and random effects models, respectively. The results indicate that the VEGF gene −1154G/A polymorphism was not associated with the risk of RCC.

### −2549I/D polymorphism and RCC

Bruyère *et al*(20) analyzed the VEGF gene −2549I/D polymorphism and RCC. The results showed that the genotype at the −2549 polymorphism exhibited a non-significant trend for an increased risk of RCC. However, the D allele was associated with a significantly increased risk [D vs. I (OR, 1.62, 95% CI, 1.04–2.53; P=0.03); DD vs. II (OR, 3.31; 95% CI, 1.13–9.64, P=0.03); DI vs. II (OR, 2.33; 95% CI, 0.85–6.43; P=0.10); DI+DD vs. II (OR, 2.64; 95% CI, 0.99–7.03, P=0.05); DD vs. II+ID (OR, 1.70; 95% CI, 0.88–3.29; P=0.11)].

### −460T/C polymorphism and RCC

**Figure 6:**
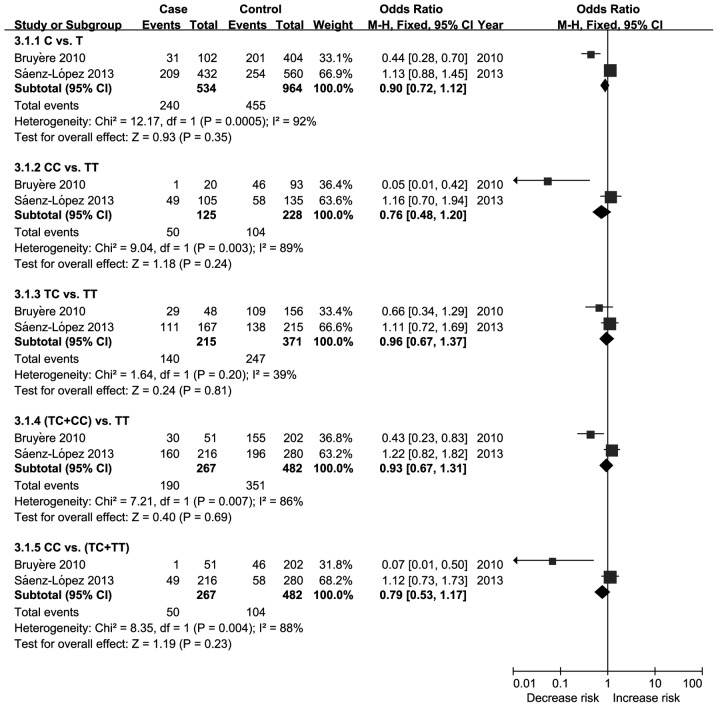
Forest plot of the association between the −460T/C polymorphism and RCC risk based on the fixed effects model. RCC, renal-cell carcinoma; CI, confidence interval.

**Figure 7:**
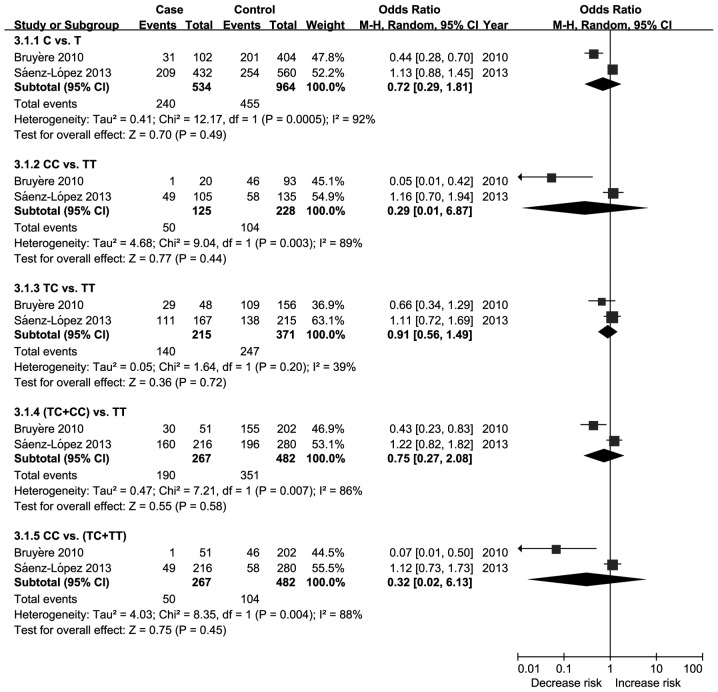
Forest plot of the association between the −460T/C polymorphism and RCC risk based on the random effects model. RCC, renal-cell carcinoma; CI, confidence interval.

A total of two studies (20,22) investigated the −460T/C polymorphism and RCC. Figs. 6 and 7 show the results of the meta-analysis based on the fixed effects and random effects models, respectively. The results indicated that the VEGF gene −460T/C polymorphism was not associated with the risk of RCC.

### 405G/C polymorphism and RCC

**Figure 8:**
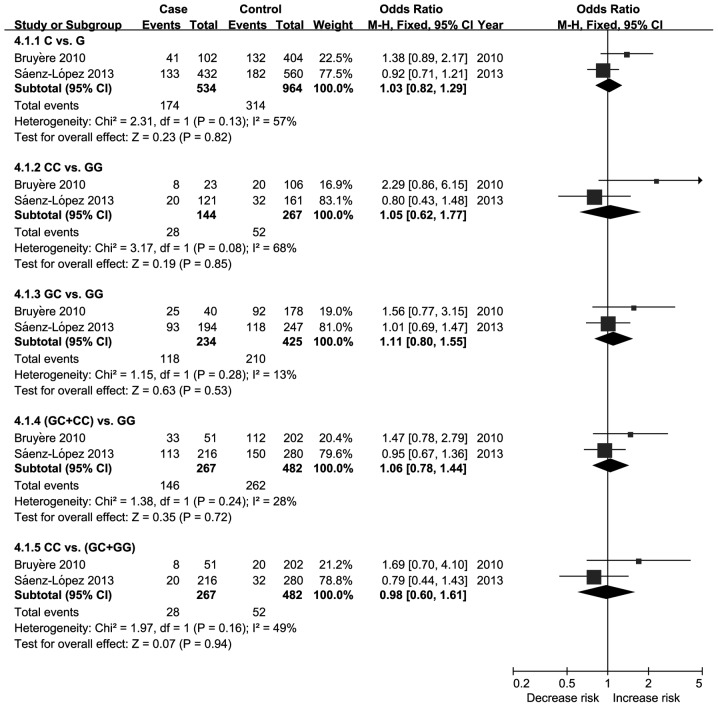
Forest plot of the association between the 405G/C polymorphism and RCC risk based on the fixed effects model. RCC, renal-cell carcinoma; CI, confidence interval.

**Figure 9:**
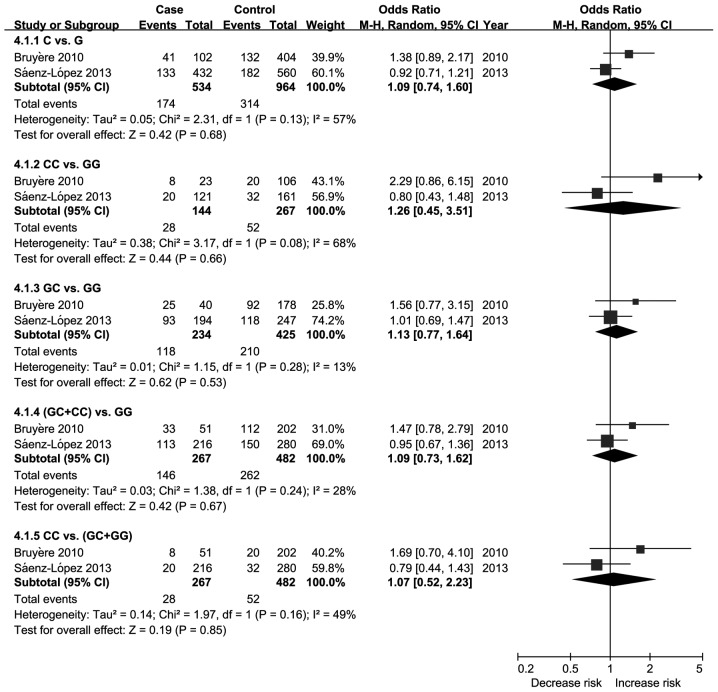
Forest plot of the association between the 405G/C polymorphism and RCC risk based on the random effects model. RCC, renal-cell carcinoma; CI, confidence interval.

A total of two studies (20,22) investigated the 405G/C polymorphism and RCC. Figs. 8 and 9 show the results of the meta-analysis based on the fixed effects and random effects models, respectively. The results indicate that the VEGF gene 405G/C polymorphism exhibited a non-significant trend for an increased risk of RCC.

### −2578C/A polymorphism and RCC

**Figure 10:**
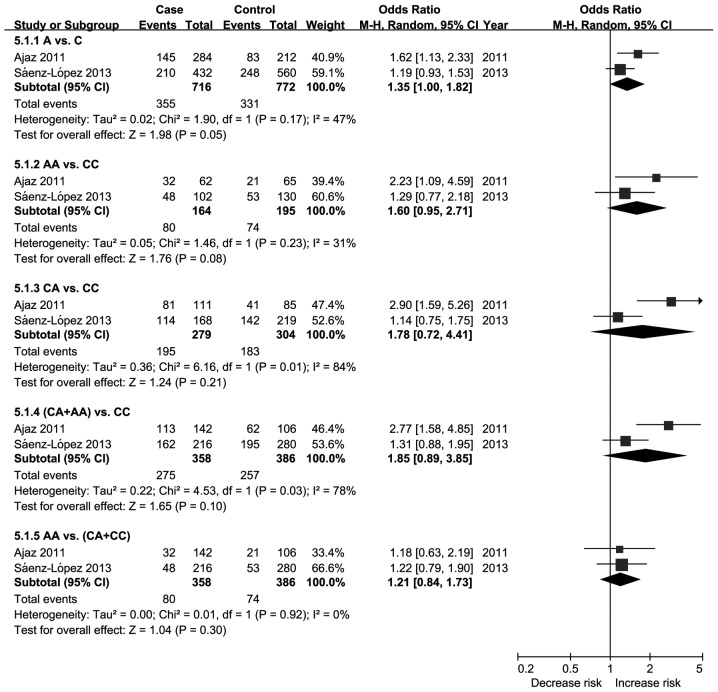
Forest plot of the association between the −2578C/A polymorphism and RCC risk based on the random effects model. RCC, renal-cell carcinoma; CI, confidence interval.

**Figure 11:**
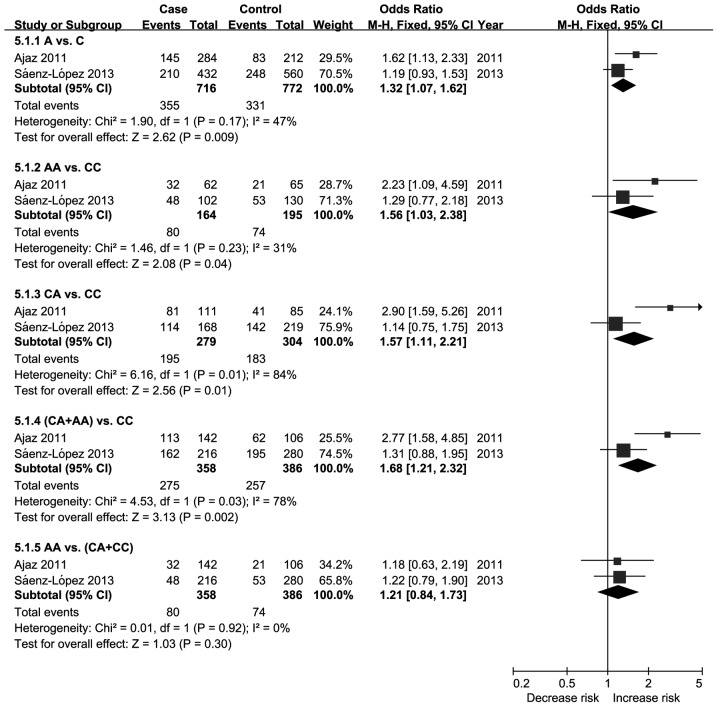
Forest plot of the association between the −2578C/A polymorphism and RCC risk based on the fixed effects model. RCC, renal-cell carcinoma; CI, confidence interval.

A total of two studies (21,22) investigated the −2578C/A polymorphism and RCC. Based on the random effects model, the results show that the genotype at the −2578C/A polymorphism exhibited a non-significant trend for a significantly increased risk of RCC, but that the A allele was associated with an increased risk of RCC (Fig. 10). However, when switched to the fixed effects model, the results show that the genotype at the −2578C/A polymorphism exhibited a significant trend for an increased risk of RCC (Fig. 11).

## Discussion

The human VEGF gene is localized in chromosome 6p21.3 (23) and comprises a 14-kb coding region organized in eight exons, which are separated by seven introns (24). Experiments have shown that increased VEGF expression is associated with tumor growth and metastasis, and the inhibition of VEGF signaling has been shown to suppress tumor-induced angiogenesis and tumor growth (25). The VEGF gene includes at least three polymorphisms that are relatively common and may affect VEGF expression. The insertion/deletion polymorphism (I/D) at the −2549 position of the promoter region and the −634G/C (rs2010963) polymorphism located in the 5′-UTR have been considered to be associated with increased VEGF expression (26,27). The 936C/T (rs3025039) polymorphism located in the 3′-UTR is associated with substantially increased serum VEGF levels (28,29). However, there are at least 30 single nucleotide polymorphisms in the VEGF gene that have been described (30). In addition, the VEGF family includes five VEGF ligands (VEGF-A, -B, -C and -D and platelet-derived growth factor), and three tyrosine kinase receptors (VEGFR-1, -2 and -3), which are involved in signaling pathways for angiogenesis and/or lymphangiogenesis (31). Therefore, an improved understanding of these markers is expected to reveal significant information with regard to the outcome and therapeutic efficacy in RCC. The present systematic review and meta-analysis was performed to investigate whether these three polymorphisms, and others, are associated with RCC.

This systematic review and meta-analysis addresses the association between eight VEGF gene polymorphisms and RCC susceptibility. Data from published studies were combined to evaluate the genetic associations between VEGF and the studied polymorphisms of the VEGF gene, namely, the 936C/T, 1612G/A, 702C/T, −1154G/A, −2549I/D, −460T/C, 405G/C and −2578C/A polymorphisms. No associations were identified between the 936C/T, 1612G/A, −1154G/A, −2549I/D, −460T/C and 405G/C polymorphisms and RCC. However, the results did reveal a significant association between the D allele of the −2549I/D polymorphism and RCC. The −2578C/A polymorphism may be associated with RCC. For the 702C/T polymorphism and RCC, the results showed that there was no polymorphism in 702C/T in either the case group or the control group. The results indicate that the −2578C/A polymorphism may be a risk factor for RCC susceptibility.

The present systematic review and meta-analysis has the following strengths. Firstly, the study is methodologically rigorous. The Q and I^2^ statistics were checked; the I^2^ statistic is more stable and is not affected as much by sample size (18). In the pooled analyses, fixed and random effects models were used to ensure the robustness of the estimates. Secondly, analysis comparisons of all five genetic models were performed, which provided enough information to detect the association. Finally, the literature search was extensive, locating all studies that had been published with regard to VEGF gene polymorphisms and RCC. Eight polymorphisms were identified, which is essential for the integrity of a comprehensive understanding of the correlation between VEGF gene polymorphisms and RCC.

This systematic review and meta-analysis has certain implications. Firstly, there was no polymorphism in 702C/T in either the RCC group or the control group. Therefore, further studies have not been required to investigate this polymorphism and RCC. In fact, since the study by Abe *et al*(15) in 2002, no subsequent studies have investigated the VEGF gene 702C/T polymorphism and RCC. Secondly, the −2578C/A polymorphism was associated with RCC risk in the fixed effects model, but heterogeneity was present and the credibility of the result was undermined. However, one of the included studies revealed an increased trend in the RCC risk and another indicated a statistically significant increased trend. Since the number and sample sizes of the two included studies were small, further studies that focus on this polymorphism are required. Thirdly, the result from the VEGF gene −2549I/D polymorphism exhibited a non-significant trend for an increased RCC risk in the DD vs. II, DI vs. II, DI+DD vs. II and DD vs. II+ID genetic models; however, the D allele was significantly associated with an increased RCC risk (OR, 1.62; 95% CI, 1.04–2.53; P=0.03). Only one study (20) detected the −2549I/D polymorphism and only two genetic models (D vs. I and DD vs. II) showed a significant difference. Further studies with regard to this polymorphism and RCC are required. Fourthly, there was no association between the 936C/T, 1612G/A, −1154G/A, −2549I/D, −460T/C and 405G/C polymorphisms and the risk of RCC, based on the fixed effects or random effects models. The meta-analysis included only three studies and further analyses are required to investigate these polymorphisms. Finally, in gene-targeted therapy (32), an assessment of the effectiveness of VEGF antibodies, including bevacizumab (33) and new VEGF inhibitor drugs for RCC are required.

However, the present results should be interpreted with caution due to the limitations apparent in this systematic review and meta-analysis. Firstly, only published studies were included in the study, therefore, publication bias may have occurred. Secondly, the number of studies and the sample sizes of each polymorphism were small, therefore, the statistical power is affected. Thirdly, the subjects in the present meta-analysis may be regarded as heterogeneous, as indicated by the heterogeneity test that made the reliable estimates should be discounted. Fourthly, this study is based on unadjusted estimates, while a more precise analysis may be performed if individual data were available. Finally, gene-gene and gene-environment interactions were not investigated due to a lack of such information in the included studies.

In conclusion, this systematic review and meta-analysis suggests that the VEGF 936C/T, 1612G/A, −1154G/A, −2549I/D, −460T/C and 405G/C gene polymorphisms are not associated with the risk of RCC. There is no 702C/T polymorphism in RCC and the −2578C/A gene polymorphism may be associated with an increased risk of RCC. Due to the limitations of the present study, further high quality case-control studies are warranted to confirm these findings.

## Figures and Tables

**Table I:** Baseline characteristics of the included studies.

		Sample size, n			
				
First author, year (ref)	Ethnicity	Case	Control	Gene polymorphism	Genotyping method
Abe *et al,* 2002 (15)	Asian	145	145	936C/T (rs3025039), 1612G/A (rs10434), 702C/T	PCR-RFLP
Ricketts *et al,* 2009 (19)	Caucasian	343	321	−1154G/A (rs1570360)	PCR
Bruyère *et al,* 2010 (20)	Caucasian	51	202	936C/T (rs3025039), −1154G/A (rs1570360),−2549I/D, −460T/C (rs833061), 405G/C (rs2010963)	PCR
Ajaz *et al,* 2011 (21)	Asian	143	106	−2578C/A (rs699947) 936C/T (rs3025039)	PCR-RFLP
Sáenz-López *et al,* 2013 (22)	Caucasian	216	216	936C/T (rs3025039), −460T/C (rs833061), −2578C/A (rs699947)	TaqMan

PCR-RFLP, polymerase chain reaction-restriction fragment length polymorphism.

**Table II:** Genotype distribution of all the included studies.

A, 936C/T (rs3025039)									

		Case				Control			
					
Reference	Location	CC	TT	CT	Source of control	CC	TT	CT	P-value for HWE
Abe 2002	3′UTR	97	41	7	HC	90	52	3	0.146
Bruyère 2010	3′UTR	29	17	1	HC	141	53	2	0.124
Sáenz-López 2013	3′UTR	156	57	2	HC	200	73	7	0.912

B, 1612G/A (rs10434)									

		Case				Control			
					
Reference	Location	GG	GA	AA	Source of control	GG	GA	AA	P-value for HWE

Abe 2002	3′UTR	113	31	1	HC	109	33	3	0.788

C, 702C/T									

		Case				Control			
					
Reference	Location	CC	CT	TT	Source of control	CC	CT	TT	P-value for HWE

Abe 2002	3′UTR	145	0	0	HC	145	0	0	/

D, −1154G/A (rs1570360)									

		Case				Control			
					
Reference	Location	GG	GA	AA	Source of control	GG	GA	AA	P-value for HWE

Ricketts 2009	PR	134	143	47	HC	146	130	38	0.281
Bruyère 2010	PR	27	17	5	HC	94	83	25	0.322

E, −2549I/D.									

		Case				Control			
					
Reference	Location	II	ID	DD	Source of control	II	ID	DD	P-value for HWE

Bruyère 2010	PR	5	28	18	HC	45	108	49	0.322

F, −460T/C (rs833061).									

		Case				Control			
					
Reference	Location	TT	TC	CC	Source of control	TT	TC	CC	P-value for HWE

Bruyère 2010	PR	19	29	1	HC	47	109	46	0.260
Sáenz-López 2013	PR	56	111	49	HC	77	138	58	0.793

G, −405G/C (rs2010963).									

		Case				Control			
					
Reference	Location	GG	GC	CC	Source of control	GG	GC	CC	P-value for HWE

Bruyère 2010	5′UTR	15	25	8	HC	86	92	20	0.522
Sáenz-López 2013	5′UTR	101	93	20	HC	129	118	32	0.528

H, −2578C/A (rs699947).									

		Case				Control			
					
Reference	Location	CC	CA	AA	Source of control	CC	CA	AA	P-value for HWE

Ajaz 2011	PR	30	81	32	HC	44	41	21	0.053
Sáenz-López 2013	PR	54	114	48	HC	77	142	53	0.388

UTR, untranslated region; PR, promoter reg.
